# Use of Nile tilapia (*Oreocromis niloticus*) processing residues in the production of pâtés with the addition of oregano (*Origanum vulgare*) essential oil

**DOI:** 10.1371/journal.pone.0296106

**Published:** 2023-12-18

**Authors:** Marcos Antonio Matiucci, Iza Catarini dos Santos, Natallya Marques da Silva, Patricia Daniele Silva dos Santos, Gislaine Gonçalves Oliveira, Stefane Santos Corrêa, Elder dos Santos Araujo, Rafaela Said, Jaqueline Ferreira Silva, Ana Paula Sartório Chambó, Talita Aparecida Ferreira de Campos, Oscar Oliveira Santos, Claudete Regina Alcalde, Maria Luiza Rodrigues de Souza, Andresa Carla Feihrmann

**Affiliations:** 1 Postgraduate Program in Food Science, Maringa State University, Maringá, Paraná, Brazil; 2 Department of Food Engineering, Maringa State University, Maringá, Paraná, Brazil; 3 Postgraduate Program in Animal Science, Maringa State University, Maringá, Paraná, Brazil; 4 Postgraduate Program in Food Engineering, Maringa State University, Maringá, Paraná, Brazil; 5 Postgraduate Program in Pharmaceutical Sciences, Maringa State University, Maringá, Paraná, Brazil; Universidad Autonoma de Chihuahua, MEXICO

## Abstract

The effect of the use of Nilo tilapia filleting residues in the production of pâtés with the addition of oregano essential oil stored for 90 days at 4 °C was evaluated. For that, 5 treatments were performed as follows: TSA—control treatment; TES with the addition of sodium erythorbate; and formulation TOE1 with 600 ppm oregano essential oil; TOE2 with 1000 ppm essential oil; and TOE3 with 1400 ppm essential oil. The pâtés showed adequate technological and physicochemical characteristics and microbiological counts within the legislation standards. No significant differences were observed in the luminosity of the pâté formulations during storage, and the addition of oil contributed to the increase in a* values and stability of b* values. Regarding the lipid and protein oxidation, TOE3 showed lower values at the end of the shelf-life. The addition of essential oil did not affect the hardness and cohesiveness of the products. The fatty acids in greater amounts in the samples were linoleic, oleic, palmitic, and stearic acids. The analysis of biogenic amines indicated that only the treatments with the highest amounts of sodium erythorbate (TES and TOE1) showed losses of spermidine. It was observed that decreasing the inclusion of sodium erythorbate and increasing the inclusion of oregano essential oil resulted in a drop in cadaverine values. A total of 46 volatile compounds were detected in the samples with the highest amount of free fatty acids and all the formulations were well accepted sensorially.

## Introduction

The increase in the demand for food products from fishing and the change in the consumer profile has increased the industry’s interest in fish. Currently, there is a growing concern with health and well-being, which has led consumers to search for quality food, with a high nutritional value, and greater convenience [1]. However, the increased demand for fish leads to a consequent increase in waste generation. Waste can reach 70% of the tilapia production, with 53.89% of the head, fins, skin, viscera, and spine, which has been discarded in an inadequate and inconsequent manner in Brazil [2].

Tons of residues are generated in the tilapia filleting, which is discarded in the environment or, at best, used for the production of meal or silage for animal nutrition [3]. However, the rational and economical use of these residues for human nutrition has been studied, such as nuggets, burgers, pâtés, sausages, bologna sausages, and other value-added products [4]. This approach can give a sustainable destination to this product, with a positive impact on the environment and economy in all stages of the fish production chain.

The pâté made from fish filleting residues has the potential to stimulate the use of waste, leading to an increase in the diversification of products offered by the fisheries market [5], some foods have been developed from tilapia filleting residues, specifically the "v" cut, showing good consumer acceptance and safety, including bread, pâté, sausages, kafta and mortadella [6–11]. Matiucci et al. [12] studied pasteurized pâtés based on Tilapia (*Oreochromis niloticus*) fish, aimed to encourage fish consumption through new processed products of quick and easy preparation. The authors reported the feasibility of food production such as pâtés using these residues. Thus, the elaboration of pâtés from fish filleting residues can be a source of alternative food, with great economic potential and social application since fish processing residues are little used despite their excellent nutritional quality.

The technical regulation of the identity and quality of pâtés establishes the maximum fat content at 32% [13], which is considered a high value susceptible to lipid oxidation, leading to a reduction in the shelf-life of the product, and changes in its physicochemical characteristics. Moreover, lipids also promote oxidation reactions, which are more pronounced in the presence of unsaturated fatty acids since the double bonds have an active center for the reaction with oxygen. This event is directly related to the exposure of the product to high temperatures or long-term storage and occurs through chemical compounds or reactive oxygen species (ROS) that break the double bonds of phospholipids in the cell membrane structure. Auto-oxidative rancidification is one of the main factors responsible for the perishing of foods, leading to the development of flavors and odors such as rancidity, decreasing the nutritional value, and limiting the shelf life of the product due to its high unsaturation [14, 15].

The use of natural antioxidants in fish-based products, such as essential oils may be a promising tool to reduce the significant oxidative potential of these products [16]. According to Feihrmann et al. [17], there has been a growing interest in the use of natural compounds in foods, replacing the synthetic antioxidants commonly used by the industry, such as sodium erythorbate. Furthermore, antioxidants can prevent the oxidative deterioration of foods.

Oregano (*Origanum vulgare*) has been widely used since ancient times in various cultures, presenting bioactive compounds, antimicrobial, antifungal, antiviral and antioxidant activity [18–22].

The oregano essential oil contains carvacrol and thymol in its composition, which is responsible for the high antioxidant and antimicrobial power of the product [23]. Several authors have reported promising results when using oregano oil in meat products for the prevention of oxidation and microbiological control [24–26].

Therefore, these compounds have the potential to replace synthetic antioxidants commonly used by the food industry, as they are good food preservatives, as well as being a product of natural origin that has health benefits and can also be used in nutraceutical products [27–29], in addition to being able to exert anti-inflammatory activity [30, 31].

Thus, the present study aimed to produce pâté using tilapia filet residues with the addition of oregano essential oil and evaluate its stability for 90 days at 4 °C for physicochemical parameters, microbiological characterization, lipid and protein oxidation, texture profile, fatty acid composition, biogenic amines, volatiles compounds, and sensory evaluation.

## Material and methods

Nile tilapia (*Oreochromis niloticus*) filleting residues (v-cut) were purchased from a tilapia slaughterhouse in the city of Rolandia/PR/Brazil, and the oregano (*Origanum vulgare*) essential oil (Quinari^®^) was obtained from a compounding pharmacy in the city of Maringa/PR/Brazil (α-pinene 1.1%, α-terpinene 0.6%, у-terpinene 1.6%, p-cymene 9.3%, linalool 0.9%, β-caryophyllene 1.7%, terpien-4ol 0.8%, thymol 4.5%, carvacrol 76.7%, others 2.7%).

### Antioxidant activity of oregano essential oil by FRAP, DPPH, and ABTS assays

The antioxidant capacity by iron reduction method was performed by mixing the sample extracts with the FRAP reagent [32]. The reagent was prepared with the combination of 0.3 M acetate buffer/10 nM TPTZ/ferric chloride 20 nM in the ratio 10:1:1 (v:v:v). To obtain the 10 nM TPTZ solution, 2,4,6-tris (2-pyridyl) -s-triazine in 40 mmol of hydrochloric acid was used. An aliquot of 90 μL of the solutions (20 μL of oregano essential oil diluted in 40 mL of ethanol) and 90 μL of solution (1g of sodium erthiorbate diluted in 10 mL of distilled water) were mixed in 270 μL of distilled water and 2.7 mL of FRAP reagent in separate test tubes, homogenized and left at 37 ºC in a water bath. The FRAP reagent was used as a blank and the absorbance reading was performed on a spectrophotometer (Model: Femto, model 700 Plus) using a wavelength of 595 nm. The sample absorbance was read after 30 minutes of incubation and the results were expressed in mg Trolox g^-1^ of sample.

The antioxidant activity of the essential oil was determined by the absence of absorbance of the 2,2-diphenyl-1-picryl hydrazyl radical (DPPH) [33]. 50 μL of oregano essential oil was diluted in 5 mL of ethanol and 0.05g of sodium erythorbate in 5 mL of water. An aliquot of 150 μL of these dilutions was used mixed with 2850 μL of methanolic DPPH solution (working solution), where DPPH was diluted in methanol until obtaining an absorbance of 1.1 nm at 515 nm in the spectrum. The sample was kept in the absence of light for 1h until the reading was performed. To read the blank, methanol was used, the absorbance was obtained in a spectrophotometer (Model: Femto, model 700 Plus) at 515 nm.

The ABTS free radical capture method [34] was evaluated using a colorimetric assay with ABTS reagents [2,2-azinobis (3-ethylbenzothiazoline-6-sulfonic acid)] and potassium persulfate (K_2_S_2_O_8_). The ABTS solution was diluted until an absorbance of 0.70 nm was reached at 734 nm. To prepare the samples in a test tube, in the dark, an aliquot of 30 μL of the solution was placed (20 μL of oregano essential oil diluted in 40 mL of ethanol) and an aliquot of 30 μL of solution (0.025 g of erythorbate of sodium diluted in 10 mL of distilled water) together with 3 mL of ABTS solution. After 6 minutes, absorbance was read at 734 nm on a spectrophotometer (Model: Femto, model 700 Plus). The results were expressed as μM trolox equivalent (TE) g^-1^ of product.

### Manufacture of the pâtés

The tilapia fillet trimmings were ground in an electric grinder (Britania, Curitiba, Brazil) five consecutive times to obtain a homogeneous batter and to reduce the spines. Then, they were weighed according to the treatments and kept refrigerated (Consul, São Paulo, Brazil) at 4 °C until the preparation of the formulations in Table 1. Five formulations were performed, as follows: control treatment (TSA) without the addition of sodium erythorbate and oregano essential oil; treatment TES with the addition of sodium erythorbate; treatment TOE1 with the addition of 600 ppm of essential oil; treatment TOE2 with the addition of 1000 ppm of essential oil; and treatment TOE3 with 1400 ppm of essential oil.

**Table 1:** Formulation of tilapia pâtés with the addition of oregano essential oil (g kg^-1^ de pâtés).

Ingredients	Treatments				
	TSA	TES	TOE1	TOE2	TOE3
Tilapia fillet trimmings	660	660	660	660	660
Margarine	113.2	113.2	113.2	113.2	113.2
Water	98.1	97.2	96.8	96.6	96.4
Ice	69.8	69.8	69.8	69.8	69.8
Textured soy protein	14.3	14.3	14.3	14.3	14.3
Salt	22.6	22.6	22.6	22.6	22.6
Monosodium glutamate	4.7	4.7	4.7	4.7	4.7
Curing salt	0.7	0.7	0.7	0.7	0.7
Maltodextrin	2.8	2.8	2.8	2.8	2.8
Modified starch	2.8	2.8	2.8	2.8	2.8
Tripolyphosphate	0.9	0.9	0.9	0.9	0.9
Cochineal carmine dye	0.1	0.1	0.1	0.1	0.1
Spices*	10	10	10	10	10
Sodium erythorbate	0.0	0.9	0.7	0.5	0.3
Essential oil**	0.0	0.0	600	1000	1400

*garlic, onion, parsley and chives dehydrated.

**expressed in ppm

The ingredients were mixed in a cutter Dadaux (Chapecó, Brazil) until the formation of emulsion and then packaged in glass jars with screw caps, with a maximum volume of 100 mL. The pâtés were pasteurized in a water bath (Mylabor, São Paulo, Brazil) at ±80 ºC for 40 minutes with the aid of an electric heater with temperature control (Mibo, Brazil), cooled in ice water at ± 4 ºC for 5 minutes, and stored under refrigeration (Consul, São Paulo, Brazil) conditions until the analysis.

### Proximate composition

The moisture, protein, and ash contents were determined according to the methodology of the Association of Official Analytical Chemists [35]. The lipid content was determined according to Bligh and Dyer [36].

### Instrumental color

The color of the samples was evaluated using Konica Minolta’s CR-400 portable colorimeter (Ramsey, USA), (D65 illuminant; 0º viewing angle, and 4 self-average). Six readings were taken in each sample, and the results were expressed by the color parameters L* (luminosity, ranging from black 0% to white 100%), a* (red-green), and b* (yellow-blue) using the CIELAB color system. The color analysis was determined on day 0 and every 15 days until day 90 of storage.

### Microbiological characterization

The microbiological analyses were performed for total coliforms as the most probable number (MPN), coagulase-positive staphylococci, and psychrotrophic bacteria counts as colony-forming units (CFU) g^-1^, and the presence of *Salmonella* sp, according to Downes and Ito [37]. All analyses were performed in triplicate. The microbiological analyses were performed on days 0, 30, 60, and 90 of storage.

### Lipid and protein oxidation

The determination of lipid oxidation was performed as described by Raharjo et al. [38]. For that, 0.5 mL of 0.5% BHT (di-tert-butyl methyl phenol) was added to a tube containing 5 g of sample. Then, 2 mL of 0.5% sulfanilamide solution was added and left to stand for 10 minutes, followed by the addition of 18 mL of 5% TCA (trichloroacetic acid). The mixture was filtered and 2 mL of the filtrate was mixed with 2 mL of 0.08 M TBA (2-thiobarbituric acid), and the reaction was carried out in a water bath (Mylabor, São Paulo, Brazil) at 80 ºC for 40 min. After the end of the reaction, absorbance readings were performed in a spectrophotometer (Agilent UV-8553, Santa Clara, USA) at 531 nm. Quantification was carried out using a standard curve (1.10^−8^ to 10.10^−8^ mol mL^-1^) of tetraethoxypropane (TEP). The results were expressed in milligrams of malonaldehyde (MDA) per kilogram of sample.

The concentration of carbonyl groups in the patês was determined using 2,4-dinitrophenylhydrazine, as described by Levine et al. [39]. Absorbance was read at 370 nm using an Evolution^™^ 300 UV-Vis spectrophotometer (Thermo Fisher Scientific, Massachusett, USA). The carbonyl group concentration was quantified using the Beer–Lambert law, *A* = *c* × *b* × ε, where *A* is the difference between sample absorbance and control absorbance, *c* is the carbonylated protein concentration, *b* is the optical path length, and ε is the molar extinction coefficient (22,000 mol cm^-1^). The results were expressed as nmol of carbonyl groups mg ^-1^ of protein.

The lipid and protein oxidation analysis were determined on day 0 and every 15 days until day 90 of storage.

### Texture profile analyses (TPA)

For the texture profile analysis (TPA), a penetration test was used at room temperature (25 ºC) using the TAXT2 texture analyzer (Stable Micro System, Surrey, United Kingdom) with a P 0.5 acrylic probe. The pre-test was carried out at 0.1 mm, the pâtés were subjected to the test with 2 cycles of penetration with 50% of the original height, the test speed was 5 mm/s with a force of 0.1N. The texture parameters hardness (N), cohesiveness, and adhesiveness (N x s) were determined.

### Fatty acids profile

Total lipids were transmethylated as described by Hartman and Lago [40]. The fatty acid methyl esters (FA) were separated on a 7890A Agilent gas chromatograph (Santa Clara, USA), coupled to a mass detector (Agilent 5975C), using an RT-x Wax Polyethylene Glycol column (30 m of length x 0.25 mm internal diameter). Helium was used as the carrier gas and the sample injection volume was 1 μL at a split ratio of 1:50. The injector and detector temperature was 250 °C, while the column temperature was 80 °C for 2 min, then increased to 235 °C at a rate of 4 °C min^-1^, remaining at this temperature for 10 min. The identification of fatty acids was performed using the NIST library (MS Search version 2.0), and the quantification was based on the relative area of methyl ester internal standard C23:0 (methyl tricosanoate). The results were expressed in g 100 g^-1^ of fatty acids. The fatty acid profile was determined on days 0 and 90.

### Analysis of biogenic amines

A derivatization free LC-MS/MS method was developed for six BAs in aquatic products and their derived products. As sample preparation procedure LLE was applied by using hexane. The polar phase was taken and diluted with 5% perchloric acid solution. the Final solution was filtered for LC-MS analysis. Because of the sizes of the column, it is possible to assume the method as an UPLC method. C18 column was used with the sizes 100 mm × 2.1 mm, 1.8 μm. Gradient eluted was preferred with a mixed solution of (0.5% formic acid) and acetonitrile. Good linearity was obtained with correlation coefficients were greater than 0.99. This method achieved higher sensitivity (from 0.1 mg kg^-1^ for tyramine, to 1.0 mg kg^-1^ for serotonin, spermidine, cadaverine, histamine and putrescine). Temperature of automatic injector was 15 °C. The injection volume was 10 μL at a flow rate of 0.3 mL min^-1^ for mobile phase and the column temperature was 40 °C. The MS analysis was in multiple-reaction monitoring (MRM) mode and the ESI was in positive ion mode. The intraday and interdays RSD values for all analytes were lower than 10% [41].

### Analysis of volatile compounds

The analysis of volatile compounds (VCs) was performed by solid phase microextraction using divinylbenzene/carboxy/polydimethylsiloxane fiber (DVB/Car/PDMS; 2 cm × 50/30 μm; Supelco^™^, Darmstadt, Germany) according to Xie et al. (2023) [42]. Thus, 5.0 ± 0.1 g of sample was placed in a 20 mL amber glass vial covered with a PTFE/silicone cap. The vial containing the sample was kept in a water bath at 35 °C for 15 min to temperature equilibrium. Then, it was maintained for 45 min for 60 °C with the fiber exposed to the headspace for the sorption of volatile compounds. Extractions were performed in duplicate for each treatment. The analysis of volatile compounds was performed in a 7890A Agilent gas chromatograph (Santa Clara, USA), coupled to a mass detector (Agilent 5975C). The fiber was thermally desorbed in the GC injection port in splitless mode at 250 °C. Helium was used as carrier gas at a flow rate of 1.2 mL min^-1^. VCs were separated on an HP-5 capillary column (30 m x 0.25 mm x 0.25 μm thick). The column temperature program started at 40 °C for 3 min, increased to 80 °C at a rate of 2 °C min^− 1^, followed by an increase of 5 °C min ^−1^ to 230 °C and remaining at this temperature for 5 min. The GC/MS interface and ionization source temperatures were maintained at 250 and 230 °C, respectively. The MS was used in electron impact ionization mode at 70 eV, and the quadrupole mass analyzer operated in scanning mode with a mass range of 35–350 m/z. The identification of the compounds was carried out by analyzing the fragmentation patterns displayed in the mass spectra, and confirmed by comparing their mass spectra with those present in the database provided by the NIST equipment (National Institute of Standards and Technology, USA), as well as their retention rates with those of known compounds, through the injection of homologous n-alkanes (C8-C20). The quantification was obtained by normalizing the areas of the compounds. The volatiles compounds analysis of the products was performed on day 30 after manufacture.

### Sensory evaluation

The research was approved by the Human Ethics Committee (Copep) of the State University of Maringa (Parana, Brazil) under protocol number 42119121.9.0000.0104. Recruitment, training, and data collection took place from April of 2021 to December of 2022. All participants gave informed written consent, which was stored by the responsible researcher. The sensory evaluation was performed through an acceptance test using a panel of 10 assessors aged 20–50 years, members of the UEM Food Engineering Laboratory team, and familiar with this type of product and terminology [14]. An acceptance test was applied using a 9-point hedonic scale, with the extremes 1 (disliked very much) and 9 (liked very much), to evaluate the attributes aroma, color, flavor, texture, appearance, and overall acceptance according to the methodology proposed by Dutcosky [43]. The assessors were provided with 40 g of sample identified with three random numbers, spread on unsalted crackers, along with water for palate cleansing. The purchase intention test was performed using a 5-point hedonic scale, in which 5 represents the maximum score "would certainly buy" and 1 represents the minimum score "would certainly not buy". The sensory evaluation of the products was performed on day 45 after manufacture.

### Statistical analyses

A complete randomized design was used, and the results were submitted to analysis of variance (ANOVA) at a 5% significance level, and Tukey’s test in case of significant differences (P<0.05), using the software Statistical Analysis System (SAS Inst. Inc., Cary, NC, USA, 2010).

## Results and discussion

### Antioxidant activity of oregano essential oil

The results of antioxidant activity of oregano essential oil are presented in Table 2. The results of the FRAP assay showed that oregano essential oil has a 65 times higher iron reducing capacity when compared to sodium erythorbate. Sarikurkcu et al. [44] studied the same type of oil and reported different values, with 133.27 μM TE g^-1^, which may be probably due to the different oil extraction methodology and the sample collection. The DPPH values of oregano essential oil were 2.4 times higher than those of sodium erythorbate. The ABTS values showed that oregano essential oil has a TEAC (Trolox Equivalent Antioxidant Capacity) 7 times higher than the synthetic antioxidant sodium erythorbate. Sarikurkcu et al. [44] reported different values from the present study, 176.41 μM TE g^-1^. Kosakowaska et al. [45] studied Greek oregano (*O*. *vulgare L*. subsp. *hirtum* (Link) Ietswaart) and common oregano (*O*. *vulgare* L. subsp.*vulgare*) and reported different antioxidant properties. Greek oregano showed 61.76%, 340.08, and 223.57 μM TE g^-1^ for DPPH, ABTS, and FRAP, respectively, while common oregano exhibited 62.01%, 342.96, and 238.13 μM TE g^-1^, respectively. According to Rice-Evans et al. [46], the phenolic compounds are responsible for neutralizing and inhibiting free radicals, which favors the use of essential oils in food composition. Concerning the quantification of phenolic compounds, the essential oil presented 6.5 times more compounds when compared to sodium erythorbate.

**Table 2:** Analysis of antioxidant activities of oregano essential oil and sodium erythorbate.

Antioxidants	FRAP (μM TE.g-^1^)	DPPH (%)	ABTS (μM TE.g-^1^)
Sodium erythorbate	21.76±2.12b	17.42±3.41b	487.41±2.91b
Oregano essential oil	1412.39±123.94a	41.73±5.46a	3496.44±150.81a
C.V. (%)	12.22	15.38	5.35
P Value	<0.01	<0.01	<0.01

a-b Mean values with different letters in the column showed significant differences between treatments (P *<* 0.05) by Tukey’s test. C.V. = coefficient of variation

### Proximate composition

The results of the proximate composition of the tilapia-trimmed pâtés with the addition of oregano essential oil are shown in Table 3. A significant difference was observed among the samples for moisture, protein, and ash contents, except for the lipid contents. The samples exhibited moisture contents from 69.01 to 71.07%, protein from 10.47 to 12.21%, lipids from 22.01 to 24.63%, and ash from 3.55 to 3.73%. These differences may be due to variations in the raw material.

**Table 3:** Chemical composition of tilapia pâtés with the addition of oregano essential oil (%).

Treatments	Parameters			
	Moisture	Protein	Lipids	Ash
TSA	70.95±0.04b	10.47±0.48b	24.43±1.92a	3.59±0.04b
TES	71.07±0.04a	11.33±0.30ab	22.01±0.06a	3.55±0.06b
TOE1	69.09±0.02d	12.21±0.26a	24.63±0.17a	3.68±0.07ab
TOE2	69.01±0.02e	12.13±0.33a	23.13±0.69a	3.73±0.04a
TOE3	69.42±0.03c	12.06±0.19a	24.63±2.86a	3.77±0.01a
C.V. (%)	1.30	6.16	6.96	2.50
P-Value	<0.01	<0.01	0.2380	<0.01

a-e Mean values with different letters in the column showed significant differences between treatments (P *<* 0.05) by Tukey’s test. C.V. = coefficient of variation.

Treatments: TSA—control treatment; TES with the addition of sodium erythorbate; and formulation TOE1 with 600 ppm of oregano essential oil; TOE2 with 1000 ppm of essential oil; and TOE3 with 1400 ppm of essential oil.

Paiva et al. [47] produced pâtés from white croaker (*Micropogonias furnieri*) and found moisture contents from 54.8 to 62.9%, protein from 6.9 to 11.6%, and lipids from 21.0 to 25.2%. Similarly, Mancera-Rodriguez et al. [48] studied pâté of white cachama (*Piaractus brachypomus*) and reported 60.20% moisture, 11.61% protein content, and 20.64% lipids. These differences may be due to the different fish species used and the different pâté formulations.

According to Brazilian legislation, pâtés should contain a maximum of 70% moisture, 32% fat, and a minimum of 8% protein [13]. Thus, only the treatments TSA and TES did not meet the legislation due to their moisture contents, which exceeded approximately 1%, while the other treatments met the parameters required by law.

### Instrumental color

S1 Fig shows the results of the color parameters of the pâtés during the storage at 4 °C. There was no significant difference for the L* values, which ranged from 67.71 to 68.28 on days 0 and 90 of storage. The values are close to those reported by other authors in fish pâté [5, 48].

Regarding the a* parameter, all treatments were similar to the control on day 0, with an average of 3.21. This behavior was not expected because the oregano oil has a greenish color, however, there was no change in the a* parameter, possibly due to the small amount used in the formulation and the effective homogenization during the manufacture of pâtés. On the last day of storage, only the treatments TOE1 and TOE3 were different from the control.

Regarding the storage time, all treatments showed an increase in a* values, except for the control treatment without the addition of antioxidant (TSA), which showed a decline in a* values from 3.33 to 2.83 on days 0 and 90. de Marins et al. [14] reported that the lipid oxidation and the oxidation of the pigment nitrosomyoglobin, resulting in metamyoglobin leads to a reduction in color. Therefore, the addition of antioxidants in the other treatments may have prevented these reactions from occurring. Different results were reported by Fernandes et al. [25], who used oregano extract in sheep sausage and reported a loss of red color in all treatments, ranging from 15.54 to 7.14. In contrast, Mancera-Rodriguez et al. [48] studied white cachama pâté and reported no changes in the initial and final a* values (P<0.05).

Regarding the b* parameter, there was no effect of oregano oil between the treatments at the beginning and end of storage, with values ranging from 16.48 and 17.82 on days 0 and 90. The b* values increased over time of storage for all treatments, indicating that the pâtés became more yellowish probably due to lipid oxidation that raises the intensity of the yellow color due to rancidity. These results agree with the lipid oxidation results, which showed an increase in values for all treatments at the end of storage. Fernandes et al. [25] found an increase in b* values for all treatments, which were less expressive for the treatments with the addition of oregano extract. However, Mancera-Rodriguez et al. [48] reported a decrease in b* values, probably due to the synthetic antioxidants used in the formulations.

Cruxen et al. [5] reported similar a* and b* values to the present study for functional fish pâtés, with a* and b* values of 2.2 and 20.18 for pâtés made with monkfish (*Oligosarcus robustus*) and 2.35 and 19.47, respectively, for pâtés made with scallop (*Loricariichthys anus*).

### Microbiological characterization

The results of the microbiological analyses are shown in S1 Table. All samples indicated that the products were fit for human consumption throughout the storage period (90 days) from a microbiological point of view. In addition to good hygienic-sanitary practices, the efficiency of heat treatment during the pasteurization of the pâtés also played an important role in this scenario. However, since there was no microbial growth for all treatments regardless of the time of storage, there is no way to attest to the antimicrobial capacity of the oregano essential oil in this experiment, once no significant difference was observed between the treatments without the addition of essential oil and the other treatments.

### Lipid and protein oxidation

**Fig 1:**
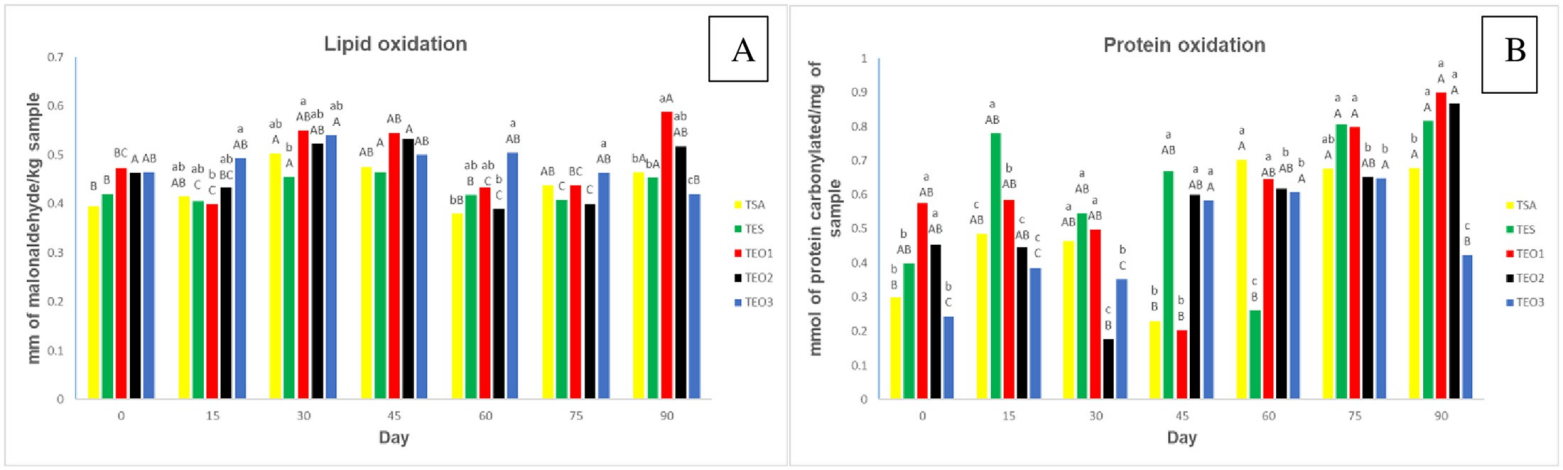
A- Lipid oxidation (mg MDA kg^-1^ sample) and B- Protein oxidation (nmol of carbonyl protein mg^-1^ sample) values of tilapia pâtés with the addition of oregano essential oil during storage time. a-d Mean values with different letters showed significant differences between treatments (P < 0.05) by Tukey’s test. A-C Mean values with different letters showed significant differences between days (P < 0.05) by Tukey’s test. Treatments: TSA - control treatment; TES with the addition of sodium erythorbate; and formulation TOE1 with 600 ppm of oregano essential oil; TOE2 with 1000 ppm of essential oil; and TOE3 with 1400 ppm of essential oil.

Lipid oxidation negatively affects the color and the sensory attributes of foods once the oxidation products can destroy or assist in the cross-linking of amino acids, making them biologically unavailable [14]. As shown in Fig 1A, there was an increase in lipid oxidation values during the storage, followed by a decrease for all treatments. No significant differences (P<0.05) were observed for lipid oxidation of the pâté formulations on day 0, which averaged 0.439 milligrams of malonaldehyde per kilogram of sample.

However, a significant difference between the treatments was observed on day 90 of storage, and the treatment TOE3 containing a higher oregano oil concentration (1400 ppm) showed the lowest lipid oxidation contents (0.420) proving the effectiveness of the oil in preventing oxidation, possibly due to the phenolic compounds present in oregano, such as carvacrol and thymol, which are known to have the antioxidant capacity [26]. According to Ariza-Nieto et al. [49], antioxidant compounds react with lipid radicals and hydroxyls converting them into more stable products. Thus, it is possible to replace the artificial antioxidant sodium erythorbate with oregano oil at a concentration of 1400 ppm, which is healthier for the consumer.

Our results corroborate with Shange et al. [26], who used oregano essential oil in black wildebeest meat (*Connochaetes gnou*). In contrast, Fernandes et al. [25], observed no difference in lipid oxidation values on day 135 of storage of sausages containing oregano extract when compared to the products without the antioxidant agent. These differences are probably due to the use of oregano extract rather than oregano oil, which may have higher antioxidant content.

According to some authors, the odor threshold of lipid oxidation is 2.5 mg MDA kg^-1^ [50], thus all treatments in the present study had acceptable values.

Regarding protein oxidation, a significant difference was observed between treatments (P<0.05) on all days studied (Fig 1B). However, on day 90 of storage, the treatment with higher oregano oil content showed the lowest protein oxidation (0.422 nmol of carbonyl protein mg^-1^ sample). The results suggest an antioxidant effect of the oregano essential oil (1400 ppm) in preventing the formation of carbonyls.

The storage time had a significant effect on protein oxidation, with an increase during the 90 days for all treatments, ranging from ±0.3937 on day 0 to ±0.736 on day 90. There is an association between the protein oxidation of pâté over time with the temperatures used in the heat treatment of the pâté. Possibly, heating leads to a degradation of myoglobin, causing the release of iron and increasing its pro-oxidant potential in cooked meat [51].

### Texture profile analysis

S2 Fig shows the results of TPA of the tilapia-trimmed pâtés. Overall, the results suggest that the addition of oregano oil to the formulations had little effect on the TPA values. On days 0 and 90 of storage, all treatments did not differ from the control for the parameter hardness. However, the storage time led to a decrease in hardness for all treatments ranging from 5.30 to 4.17 on days 0 and 90. Opposite results were reported by Lago et al. [52], who found an increase in hardness in frozen tilapia sausages during the storage, probably due to emulsion instability, with water separation and an increase in firmness of the product, which was not observed in the present study.

The cohesiveness was not affected by the addition of oregano oil and the storage time. Regarding the parameter adhesiveness, there was no difference between the control and the other treatments on days 0 and 90 of storage. All treatments showed a decrease in adhesiveness values until day 60, with an increase after this period, except for the treatment TOE2.

### Fatty acid profile

The fatty acid profile of the pâtés is shown in Table 4. The linoleic fatty acid was found in the highest amount, followed by oleic, palmitic, and stearic acids. The pâtés exhibited SFA contents between 33.25%±1.51 to 40.25%±0.40. Regarding MUFA, the contents ranged from 27.25% ±0.13 to 35.91±0.14, and the treatments TES and TOE1 showed an increase in MUFA values between days 0 and 90. Oleic acid was the monounsaturated fatty acid found in the highest concentration, similar to the findings of Mancera-Rodriguez et al. [48]. The PUFA contents (32.46% ±0.15–35.30%±0.13) were higher than MUFA contents, possibly due to the high concentration of linoleic acid in the pâtés. No degradation (P<0.05) of these fatty acids was observed for all treatments throughout the storage. In addition, no significant differences (P<0.05) were observed for PUFA for all treatments over time, indicating that although they are more susceptible to oxidation, there was no oxidation of the products.

**Table 4:** Fatty acids profile of of tilapia pâtés with the addition of oregano essential oil during storage time (g 100 g^-1^ of fatty acids).

Fatty acid	Day	Treatments					C.V. (%)	P Value
		TSA	TES	TOE1	TOE2	TOE3		
Capric acid	0	0.21±0.00aA	0.20±0.0 aA	0.24±0.02aA	0.29±0.02aA	0.25±0.01aA	15.37	0.5022
C10:0	90	0.14±0.01aA	0.12±0.01aA	0.22±0.01aA	0.21±0.00aA	0.25±0.01aA	29.35	0.1229
C.V. (%)		24.01	30.03	7.70	19.57	4.62		
P Value		0.2196	0.1638	0.7194	0.1238	1.0000		
Lauric acid	0	3.21±0.01aA	3.38±0.09aA	3.57±0.29aA	4.03±0.09aA	3.17±0.01aA	10.36	0.1943
C12:0	90	2.82±0.06aA	2.67±0.03aA	3.42±0.00aA	3.14±0.01aB	3.33±0.05aA	9.96	0.2525
C.V. (%)		7.78	13.79	7.12	14.4	3.15		
P Value		0.3061	0.0762	0.6931	0.0314	0.6739		
Myristic acid	0	2.24±0.02aA	2.33±0.01aA	2.81±0.03aA	2.51±0.03aA	2.81±0.01aA	9.95	0.1277
C14:0	90	2.18±0.02aA	2.22±0.02aA	2.32±0.01aA	2.35±0.03aA	2.75±0.00aA	9.00	0.2476
C.V. (%)		1.86	2.81	11.08	4.08	1.27		
P Value		0.8342	0.6899	0.0771	0.5447	0.8193		
Pentadecanoic acid	0	0.08±0.01aA	0.07±0.01aA	0.05±0.01aA	0.09±0.01aA	0.12±0.02aA	36.16	1.0000
C15:0								
	90	1.71±0.01aA	1.72±0.02aA	1.46±0.02aA	1.50±0.02aA	1.64±0.02aA	7.07	0.9972
C.V. (%)		105.77	107.05	108.59	103.11	99.76		
P Value		0.0843	0.0817	0.1304	0.1304	0.1062		
Palmitic acid	0	16.30±0.08bA	16.23±0.46bA	20.88±0.45aA	17.94±0.40abA	17.66±0.58abA	10.3	0.0048
C16:0	90	17.79±0.24aA	18.00±0.04aA	16.71±0.46B	17.89±0.01aA	15.99±1.47aA	6.32	0.3176
C.V. (%)		5.21	6.35	12.97	1.81	9.56		
P Value		0.1951	0.1302	0.0018	0.968	0.1518		
Palmitoleic acid	0	1.70±0.02abA	1.68±0.08abA	1.46±0.08bA	1.84±0.05abA	2.13±0.12aA	13.9	0.0093
C16:1								
	90	1.66±0.02aA	1.62±0.01aA	1.74±0.01aA	1.67±0.01aA	1.74±0.05aB	3.29	0.9216
C.V. (%)		1.71	4.34	10.75	6.08	12.78		
P Value		0.8247	0.7046	0.0969	0.3048	0.0239		
Margaric acid	0	0.53±0.01aA	0.52±0.01aA	0.43±0.01aA	0.14±0.01bA	0.16±0.01bA	51.29	0.0171
C17:0	90	0.43±0.42aA	0.31±0.01aA	0.23±0.02aA	0.24±0.02aA	0.29±0.02aA	26.03	0.5810
C.V. (%)		12.45	29.35	36.42	30.29	34.62		
P Value		0.4607	0.1327	0.1415	0.4830	0.3406		
Cis-10-heptadecenoic acid C17:1	0	0.13±0.01aA	0.14±0.01aB	0.22±0.01aA	0.12±0.01aA	0.12±0.03aA	29.1	0.8746
	90	0.35±0.01aA	0.46±0.02aA	0.17±0.01aA	0.18±0.00aA	0.33±0.02aA	39.68	0.0735
C.V. (%)		54.83	63.33	14.31	22.4	57.01		
P Value		0.0536	0.0085	0.6812	0.6161	0.0638		
Estearic acid	0	11.20±0.09aA	11.20±0.34aA	11.93±0.40aA	9.89±0.33aA	11.80±0.47aA	7.54	0.4887
C18:0	90	9.78±0.02aA	10.51±0.04aA	8.67±0.17aB	9.23±0.08aA	8.73±0.04aA	7.91	0.5316
C.V. (%)		8.31	4.51	18.59	4.90	17.64		
P Value		0.2412	0.5725	0.0163	0.5918	0.0224		
Oleic acid	0	24.64±0.26abA	24.88±0.45abA	23.17±0.27bB	24.94±0.11abA	28.03±0.19aA	6.76	0.0292
C8:1	90	27.21±0.11aA	27.02±0.12aA	27.02±0.21aA	27.25±0.08aA	26.26±0.06aA	1.49	0.9420
C.V. (%)		5.81	4.98	8.92	5.13	3.82		
P Value		0.0693	0.1251	0.0106	0.0999	0.1979		
Vaccenic acid	0	1.62±0.01aA	1.28±0.05aA	1.60±0.10aA	1.35±0.03aA	1.23±0.10aA	13.08	0.2176
C18:1 n-7	90	1.55±0.01aA	1.31±0.01aA	0.96±0.03bB	0.93±0.02bA	1.48±0.03aA	21.97	0.0190
C.V. (%)		2.69	3.31	29.66	21.71	12.23		
P Value		0.7328	0.8643	0.0062	0.0519	0.2332		
Linoleic acid	0	30.87±0.03abA	31.46±0.63abA	33.08±0.18aA	31.46±0.24abA	29.46±0.48bB	4.13	0.0149
C18:2	90	31.15±0.11aA	30.12±0.10aA	32.30±0.01aA	31.67±0.23aA	32.50±0.10aA	2.90	0.0920
C.V. (%)		0.60	3.04	1.46	0.94	5.80		
P Value		0.7498	0.1461	0.3851	0.8141	0.0035		
GLA (Gamma linolenic acid)	0	0.29±0.01aA	0.31±0.01aA	0.26±0.02abA	0.10±0.01baA	0.15±0.00abA	39.74	0.0053
	90	0.16±0.01aB	0.16±0.01aB	0.17±0.01aA	0.17±0.01aA	0.19±0.01aA	9.22	0.9717
C.V. (%)		33.75	37.74	25.62	29.15	16.44		
P Value		0.0302	0.0145	0.1183	0.2500	0.4204		
Linolenic acid C18:3	0	2.26±0.03aA	2.61±0.04aA	2.99±0.03aA	2.88±0.02aA	3.62±0.05aA	16.6	0.0775
	90	2.40±0.01aA	2.15±0.01aA	2.38±0.04aA	2.19±0.03aA	2.56±0.02aB	6.88	0.8797
C.V. (%)		3.95	11.11	13.26	15.76	19.67		
P Value		0.7383	0.3183	0.1866	0.1383	0.0309		
Gondoic acid C20:1	0	0.72±0.02aA	0.71±0.01aB	0.81±0.01aA	0.51±0.01aA	0.38±0.01aA	26.9	0.9926
	90	3.45±0.01aA	5.51±0.04aA	4.29±0.05aA	4.28±0.02aA	3.88±0.01aA	16.97	0.9020
C.V. (%)		75.78	89.28	78.81	91.07	95.12		
P Value		0.2245	0.0415	0.1272	0.1006	0.1247		
Arachidonic acid	0	0.24±0.00abA	0.23±0.01abA	0.29±0.01aA	0.22±0.01bA	0.23±0.00abB	11.91	0.0276
C20:4								
	90	0.26±0.01aA	0.26±0.01aA	0.25±0.01aA	0.24±0.01aA	0.28±0.02aA	6.94	0.461
C.V. (%)		6.9	9.14	9.56	5.53	12.65		
P Value		0.2601	0.1221	0.0808	0.4933	0.0335		
Behenic acid	0	0.33±0.02aA	0.31±0.01aA	0.37±0.02aA	0.12±0.01aA	0.13±0.01aA	45.33	0.0658
C22:0	90	0.40±0.01aA	0.42±0.02aA	0.31±0.02aA	0.30±0.02aA	0.28±0.01aA	18.13	0.5480
C.V. (%)		11.79	17.21	11.56	51.08	43.4		
P Value		0.4977	0.3137	0.5602	0.0941	0.1571		
DHA	0	0.23±0.01aA	0.24±0.01aA	0.23±0.02aA	0.27±0.01aA	0.21±0.02aA	10.79	0.1186
C22:6	90	0.27±0.01aA	0.19±0.01bB	0.21±0.01abA	0.22±0.01abB	0.24±0.00abA	13.87	0.0166
C.V. (%)		10.33	16.33	7.85	13.62	9.97		
P Value		0.0852	0.0145	0.5	0.0229	0.2674		
SFA	0	34.12±0.00bA	34.23±0.03bA	40.25±0.40aA	34.98±0.87bA	36.08A±1.12bA	6.88	0.0037
	90	35.23±0.52aA	34.95±0.11aA	33.31±0.13aB	34.84±0.11aA	33.25±1.51aA	3.9	0.2859
C.V. (%)		2.04	2.86	10.91	1.47	6.46		
P Value		0.4519	0.2499	0.0002	0.9237	0.0677		
MUFA	0	28.79±0.35aA	28.67±0.46aB	27.25±0.13aB	28.75±0.05aA	31.87±0.42aA	5.58	0.7000
	90	34.21±0.11aA	35.91±0.14aA	34.17±0.19aA	34.29±0.14aA	33.68±0.04aA	2.35	0.9650
C.V. (%)		9.97	12.98	13.01	10.16	3.35		
P Value		0.1133	0.0402	0.0488	0.1061	0.584		
PUFA	0	33.36±0.02abA	34.32±0.84abA	36.29±0.28aA	34.61±0.38abA	33.29±0.41bA	3.52	0.0414
	90	33.83±0.11aA	32.46±0.15aA	34.88±0.04aA	34.08±0.37aA	35.30±0.13aA	3.07	0.0759
C.V. (%)		0.84	3.54	2.33	1.27	3.54		
P Value		0.6267	0.0708	0.1611	0.5842	0.0529		
ω6/ω3	0	13.69±0.32aA	12.06±0.60aA	11.08±0.10aA	10.92±0.01aA	8.15±0.23aB	17.19	0.089
	90	12.96±0.18aA	13.97±0.02aA	13.60±0.37aA	14.46±0.13aA	12.67±0.09aA	5.22	0.857
C.V. (%)		13.32	13.02	12.34	12.69	10.41		
P Value		0.6903	0.3086	0.1856	0.0703	0.0252		

a-b Mean values with different letters showed significant differences between treatments (P *<* 0.05) by Tukey’s test. A-B Mean values with different letters showed significant differences between days (P *<* 0.05) by Tukey’s test. C.V. = coefficient of variation.

Treatments: TSA—control treatment; TES with the addition of sodium erythorbate and formulation TOE1 with 600 ppm of oregano essential oil; TOE2 with 1000 ppm of essential oil; and TOE3 with 1400 ppm of essential oil.

The ω6/ω3 ratio did not differ between treatments, except for the treatment TOE3, which showed a loss of 35.83%, with values ranging from 13.69±0.32 to 8.15±0.23, above the value of 2.25 found by Mancera-Rodriduez et al. [48]. Saltwater fish typically have a higher proportion of n-3 fatty acids, however, freshwater fish such as tilapia contains a higher n-6/n-3 ratio when compared to marine fish [53].

### Analysis of biogenic amines

The accumulation of biogenic amines in food products can be a good indicator of spoilage. Table 5 shows that there was no significant difference (P>0.05) between treatments at the end of 90 days in relation to spermidine.

**Table 5:** Biogenics amines in tilapia pâtés with the addition of oregano essential oil (mg kg^-1^ pâtés).

Biogenics amines	Day	Treatments					C.V. (%)	P- Value
		TSA	TES	TOE1	TOE2	TOE3		
Spermidine	0	3.15±0.00aA	3.17±0.00aA	3.22±0.03aA	3.16±0.01aA	3.14±0.00aA	9.09	0.1793
	90	2.73±0.01aA	2.23±0.00aB	2.46±0.01aB	2.92±0.01aA	2.19±0.00aA	11.81	0.1319
C.V. (%)		7.64	19.18	14.67	4.33	7.58		
P- Value		0.2149	0.0072	0.0265	0.4633	0.3252		
Cadaverine	0	0.13±0.00bA	1.06±0.04 aA	0.26±0.00bA	0.28±0.00bA	0.14±0.00bA	96.19	>0.01
	90	0.66±0.02abA	1.52±0.02aA	0.34±0.02bA	0.42±0.01abA	0.18±0.00bA	79.00	0.0128
C.V. (%)		73.30	19.88	14.17	21.98	13.69		
P- Value		0.1751	0.2310	0.8407	0.7138	0.9164		
Putrescine	0	0.11±0.00 aA	0.08±0.00aA	0.15±0.00aA	0.14±0.01aB	0.07±0.00aA	28.31	0.8275
	90	0.20±0.01abA	0.23±0.00abA	0.29±0.00abA	0.38±0.00aA	0.13±0.01bA	36.63	0.0388
C.V. (%)		30.60	53.00	35.72	52.13	31.07		
P- Value		0.2792	0.0671	0.0861	0.0047	0.4762		

a-b Mean values with diferents letters showed significant differences between treatments (P *<* 0.05) by Tukey’s test. A-B Mean values with diferents letters showed significant differences between days (P *<* 0.05) by Tukey’s test. C.V. = coefficient of variation.

Treatments: TSA—control treatment; TES with the addition of sodium erythorbate; and formulation TOE1 with 600 ppm of oregano essential oil; TOE2 with 1000 ppm of essential oil; and TOE3 with 1400 ppm of essential oil.

On the other hand, for cadaverine values, it was noticed that the treatment elaborated only with sodium erythorbate (TES), presented a relatively high initial and final value when compared to the other treatments. It is possible that exposure to high temperature during the moment of pasteurization, added to sodium erythorbate, contributed to this significant increase in cadaverine values. It is possible to observe that as sodium erythorbate decreases and oregano essential oil increases between treatments (TES, TOE1, TOE2 and TOE3), there is a decrease in cadaverine values, indicating that this substitution has beneficial potential for human health.

Regarding putrescin, there was no difference between the treatments at time zero (P>0.05), indicating that the high temperature had no effect on this biogenic amine, however at 90 days of storage, the TOE2 treatment showed an increase in its putrescine content when compared to the initial period, day zero. However, the treatment with the highest inclusion of oregano essential oil, TOE3 presented the lowest average among the treatments during the final shelf-life period (90 days), presenting an effect similar to that which occurred with cadaverine. Thus, further studies with oregano essential oil in tilapia-based pâtés are suggested, because in the case of biogenic amines, the results have shown promise, since the raw material was a filleting residue, and not the main raw material (fillet).

Although the biogenic amines, putrescine and cadaverine are the most common in foods, it is important to prevent their accumulation, as they potentiate the adverse effects of other biogenic amines, favoring their adsorption or interfering with the detoxification system, another important factor is that these two biogenic amines can react with nitrites, producing nitrosamines [54]. All pâté samples analyzed showed levels below the limit for the biogenic amines evaluated [55].

The biogenic amines: serotonin, tyramine, and histamine were below the detection limits for all treatments, regardless of the time (days 0 and 90) analyzed.

### Volatile compounds

An average of 46 volatile compounds divided into alkanes, esters, aromatics, acids, aldehydes, alcohols and ketones were identified. As expected, the samples with the addition of oregano oil showed the compounds carvacrol, thymol, α-pinene, у-terpinene, cymene, linalool and caryophyllene, which did not occur with the TSA and TES samples that did not contain oil. As seen in Table 6, the compounds found in greater amounts were free fatty acids such as oleic acid methyl ester, palmitic acid methyl ester, linoleic acid methyl ester. According to Wang et al. [56] the increase in the values of free fatty acids in fish products correlates with the values of lipid oxidation, and the greater the lipid oxidation, the greater the content of free fatty acids. This corroborates our work because the analysis of compounds was performed on pâté samples after 30 days of storage where the values of lipid oxidation showed an increase in relation to the day of manufacture. Likewise, Xie et al. [42] when evaluating the effect of freezing on crisp Nile tilapia fillets, concluded that at the end of 120-day storage there was a significant increase in the levels of free fatty acids from lipid oxidation.

**Table 6:** Volatile compounds in tilapia pâtés with the addition of oregano essential oil (%).

Compounds		RT	Treatments					C.V. (%)	P Value
			TSA	TES	TOE1	TOE2	TOE3		
Alkanes	Pentane	2.48	0.35±0.04c	0.75±0.05b	0.29±0.04c	0.94±0.05b	1.13±0.03a	6.67	>0.01
	Hexamethyl cyclotrisiloxane	4.02	0.35±0.04c	0.63±0.05b	0.91±0.02a	0.43±0.02bc	0.63±0.11b	9.71	>0.01
	Octamethyl-cyclotrisiloxane	4.06	0.14±0.02b	0.20±0.06ab	0.12±0.03b	0.45±0.13a	0.24±0.02ab	30.49	0.0269
	Phenyl pentamethyl-disiloxane	7.71	0.37±0.08b	0.30±0.04b	0.58±0.03ab	1.06±0.23a	0.96±0.18a	20.79	>0.01
	Undecane	9.18	1.37±0.06a	1.48±0.20a	1.17±0.20a	1.53±0.18a	1.73±0.02a	13.61	0.2140
	Decamethyl cyclopentasiloxane	13.38	0.27±0.08a	0.25±0.07ab	0.15±0.01bc	0.12±0.04bc	0.10±0.01c	20.52	0.0133
	3,5 dibutoxy 1,1,1,7,7,7 hexamethyl-3,5 bis tetrasiloxane	20.80	0.47±0.08a	0.20±0.07bc	0.10±0.01c	0.39±0.04ab	0.24±0.02bc	18.51	>0.01
	Tetradecamethyl cycloheptasiloxane	23.00	0.24±0.04b	0.10±0.02c	0.09±0.01c	0.07±0.01c	0.33±0.03a	14.08	>0.01
Esters	Triethyl citrate	27.17	0.530.03±b	0.32±0.01b	0.99±0.12a	0.55±0.11b	0.350.05±b	17.35	>0.01
	Cyclopentaneundecanoic acid methyl ester	28.54	0.94±0.06ab	0.24±0.04d	1.01±0.02a	0.82±0.04b	0.41±0.06c	6.12	>0.01
	Miristic acid methyl ester	28.55	3.10±0.01a	1.72±0.06c	2.35±0.06b	1.44±0.30c	0.71±0.01d	7.66	>0.01
	Citronellic acid	31.09	0.93±0.08a	1.23±0.17a	0.60±0.51a	0.23±0.02a	0.15±0.01a	53.30	0.0871
	Methyl palmitoleate	32.47	2.38±0.39a	1.26±0.02bc	1.65±0.11ab	0.91±0.12bc	0.86±0.08c	14.08	0.0028
	Palmitic acid methyl ester	32.91	6.95±0.56c	11.93±0.55a	9.79±0.24b	6.63±0.11c	7.47±0.52c	5.28	>0.01
	Linoleic acid methyl ester	36.28	11.46±0.34ab	14.50±1.34a	10.05±0.18b	14.50±1.19a	12.25±0.49ab	6.80	0.0123
	Oleic acid methyl ester	36.42	20.80±0.98c	26.32±0.78a	22.11±0.34bc	24.20±1.12abc	25.94±1.36ab	4.15	0.094
	Estearic acid methyl ester	36.93	1.66±0.17b	1.21±0.01b	1.47±0.11b	4.01±0.35a	3.64±0.17a	8.49	>0.01
Aromatics	Alfa pinene	8.27	N.D.	N.D.	0.12±0.01c	0.37±0.07b	0.55±0.01a	12.01	>0.01
	o -cymene	9.28	N.D.	N.D.	0.37±0.07c	0.90±0.03b	3.10±0.17a	7.39	>0.01
	Y terpinene	10.37	N.D.	N.D.	0.10±0.01b	0.14±0.02b	0.88±0.05a	8.77	>0.01
	Beta linalool	11.80	N.D.	N.D.	0.10±0.01b	0.20±0.06b	0.73±0.06a	11.76	>0.01
	Tymol	17.91	N.D.	N.D.	0.52±0.06a	0.64±0.05a	0.76±0.10a	11.58	0.0987
	Carvacrol	18.26	N.D.	N.D.	1.33±0.16b	2.43±0.31b	4.40±0.57a	14.28	0.0094
	Caryophyllene	21.02	N.D.	N.D.	0.04±0.01c	0.12±0.01b	0.58±0.06a	15.56	>0.01
Acids	Hexanoic acid	11.02	0.74±0.05b	0.90±0.04b	1.61±0.04a	0.35±0.01c	0.11±0.01d	5.42	>0.01
	Undecanoic acid	23.71	2.58±0.06b	3.35±0.13a	1.72±0.01c	2.72±0.30b	2.59±0.06b	5.80	>0.01
	Isocitric acid	26.58	0.24±0.01b	0.47±0.06a	0.15±0.01bc	0.10±0.01c	0.56±0.01a	10.15	>0.01
	2,6 diaminopimelic acid	26.80	0.70±0.04a	0.24±0.03cd	0,09±0.02d	0.34±0.04bc	0.48±0.06b	10.99	>0.01
	Adipic acid	27.16	1.24±0.04a	1.57±0.02a	0.56±0.47a	0.86±0.06a	0.51±0.05a	31.72	0.0638
	Pthalic acid	31.76	0.24±0.02ab	0.15±0.01bc	0.05±0.00c	0.36±0.06a	0.24±0.03ab	21.32	>0.01
	10-undecenoic	33.71	0.34±0.04b	0.88±0.01a	0.40±0.05b	0.90±0.04a	0.84±0.01a	5.73	>0.01
	Benzoic acid	38.35	1.17±0.09c	0.87±0.09d	1.95±0.02a	1.62±0.05b	1.10±0.08cd	5.37	>0.01
Aldehydes	6-nonenal	11.82	0.41±0.01d	1.09±0.08a	0.90±0.01b	0.60±0.04c	0.90±0.03b	6.67	>0.01
	Benzaldehyde	12.43	1.60±0.17c	3.79±0.16a	2.65±0.03b	2.95±0.01b	1.01±0.08d	4.55	>0.01
	Decanal	14.93	0.24±0.01a	0.12±0.04b	0.06±0.01b	0.10±0.01b	0.13±0.0.01b	17.54	>0.01
	Heptadecenal	23.41	0.14±0.04b	0.32±0.01a	0.11±0.01b	0.02±0.00c	0.08±0.01bc	16.25	>0.01
	Undecanal	28.32	0.18±0.04a	0.05±0.01b	0.09±0.01ab	0.13±0.01ab	0.17±0.03a	20.08	0.0131
Alcohols	Terpinen-4-ol	14.11	1.35±0.11	1.17±0.14	1.24±0.18	1.27±0.04	1.32±0.01	8.93	0.5955
	3-methyl-1 -butanol	14.95	0.11±0.03b	0.20±0.02ab	0.27±0.06a	0.23±0.03ab	0.15±0.01ab	18.68	0.0373
	10-chloro-1-decanol	15.00	0.17±0.04bc	0.08±0.01c	0.40±0.01a	0.24±0.04b	0.20±0.03b	12.39	>0.01
	8-chloro 1 octanol	21.92	0.04±0.01b	0.03±0.01b	0.11±0.02a	0.04±0.01b	0.08±0.01ab	22.59	>0.01
	(z) 4-decen-1-ol	22.53	0.21±0.04bc	0.12±0.04c	0.44±0.04a	0.25±0.04bc	0.29±0.03b	14.29	>0.01
	E11,13-tetradecaqdien-1-ol	22.59	0.21±0.01ab	0.24±0.03a	0.08±0.02cd	0.03±0.01d	0.14±0.02bc	13.95	>0.01
	Hexadecanol	31.92	0.76±0.15b	0.80±0.17ab	0.49±0.08c	0.92±0.18a	0.82±0.15ab	4.06	>0.01
Ketones	Spironolactone	16.53	0.43±0.02c	0.32±0.03d	0.71±0.01a	0.42±0.01c	0.60±0.04b	4.88	>0.01
	Furanone	19.55	0.09±0.01a	0.05±0.01b	0.02±0.01c	0.03±0.00bc	0.05±0.01b	14.37	>0.01

a-d Mean values with different letters showed significant differences between treatments (P *<* 0.05) by Tukey’s test. C.V. = coefficient of variation.

RT: Retention time. N.D. no detected

Treatments: TSA—control treatment; TES with the addition of sodium erythorbate; and formulation TOE1 with 600 ppm of oregano essential oil; TOE2 with 1000 ppm of essential oil; and TOE3 with 1400 ppm of essential oil.

In addition, other compounds originating from lipid oxidation such as alcohols, ketones and aldehydes were detected in all samples, with greater amounts being eicosanol, spironolactone and benzaldehyde, respectively. Regarding the alkanes, the compounds present in greater quantities in the pâté samples were in descending order the undecane, pentane and phenyl pentamethyl-disiloxane.

### Sensory evaluation

**Fig 2:**
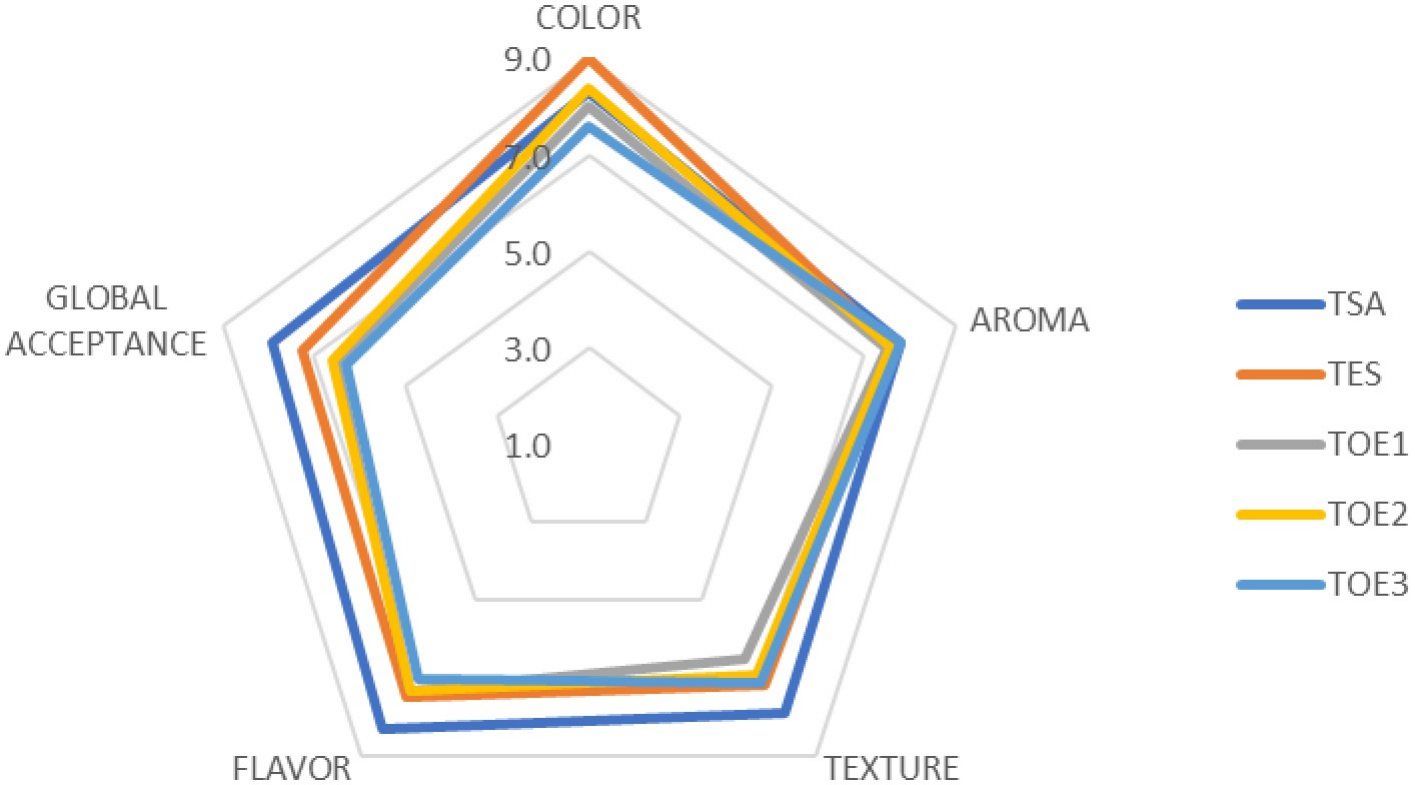
Sensory analysis of tilapia pâtés with the addition of oregano essential oil. Treatments: TSA - control treatment; TES with the addition of sodium erythorbate; and formulation TOE1 with 600 ppm of oregano essential oil; TOE2 with 1000 ppm of essential oil; and TOE3 with 1400 ppm of essential oil.

Fig 2 shows the spider diagram of the sensory attributes of the pâtés. There was no significant difference (P<0.05) among the treatments for the attributes overall impression, color, aroma, and texture. According to Dutcosky [43], the attribute color was well accepted by the assessors, classified as "liked very much", while the attributes aroma, texture, and overall impression were classified as "liked moderately". Concerning the attribute flavor, the sample with the addition of sodium erythorbate was similar to the control, with scores of 7.5 and 8.3, respectively. On the other hand, the samples with the addition of oregano oil (TOE1, TOE2, and TOE3) were different from the other treatments, with mean scores of 7.3, 7.3, and 7.1, respectively. These results suggest that the oregano oil influenced the flavor of the pâtés. All the samples were scored above 7.0, thus they were all acceptable for the attribute flavor. These results indicate that the pâtés made from tilapia filleting residues were attractive to consumers. No significant differences were observed between the treatments for the purchase intention (P<0.05), with average scores of 3.7, showing that the assessors "might buy, might not buy" the product.

Paiva et al. [47] studied pâté made with whitemouth croaker (*Micropogonias furnieri*) muscle and found sensory scores close to this study, with values ranging from 6.43 to 8.28, and 7.03 to 7.62 for color and texture, respectively. However, the attribute flavor scored from 6.62 to 8.28 while the overall acceptance scored from 6.87 to 8.15. Matiucci et al. [12] reported lower sensory scores and purchase intention of pâtés made from tilapia filleting residues, with values of 6.24. 6.4, 6.25, and 6.41, for the attributes color, aroma, texture, and flavor, respectively.

## Conclusions

The tilapia-trimmed pâtés with the addition of oregano essential oil met the technological, physicochemical, and microbiological standards throughout the 90-day storage period at 4 °C. Regarding instrumental color analysis, there was no significant difference in luminosity, however the essential oil influenced the a* and b* coordinates. The addition of 1400 ppm oregano oil provided a decrease in lipid and protein oxidation, probably due to the antioxidant compounds present in the oil. The TPA analysis showed that hardness was not affected by the replacement of sodium erythorbate with oregano essential oil, but only the TOE3 treatment remained stable throughout the 90-day period, while cohesiveness did not differ between the treatments regardless of the time evaluated, but adhesiveness varied according to the treatments. Linoleic and oleic fatty acids were found in higher quantities in the pâtés. The decrease in sodium erythorbate concomitant with the increase in oregano essential oil resulted in a decrease in cadaverine values, indicating that this decrease may be beneficial to human health. A total of 46 volatile compounds were identified, with the values of aromatic volatile compounds increasing as the inclusion of essential oil increased. All the treatments were well accepted in the sensory evaluation, thus it is possible to elaborate tilapia-trimmed pâtés with oregano oil with promising results, once besides presenting an excellent nutritional and sensory profile, it can contribute to diminishing the environmental impacts, valorize the aquaculture sector, and diversify the commercialized products.

## Supporting information

S1 Fig Color instrumental (L*, a* and b*) of tilapia pâtés with the addition of oregano essential oil during storage time. (TIF)

S2 Fig Texture profile analysis of tilapia pâtés with the addition of oregano essential oil during storage time. (TIF)

S1 Table Microbiological analysis of tilapia pâtés with the addition of oregano essential oil. (DOCX)
