# Supplementary figures and images for: Use of Nile tilapia (Oreocromis niloticus) processing residues in the production of pâtés with the addition of oregano (Origanum vulgare) essential oil
Source: PLoS One. 2023 Dec 18;18(12):e0296106. doi: 10.1371/journal.pone.0296106 (PMC10727447; doi:10.1371/journal.pone.0296106)

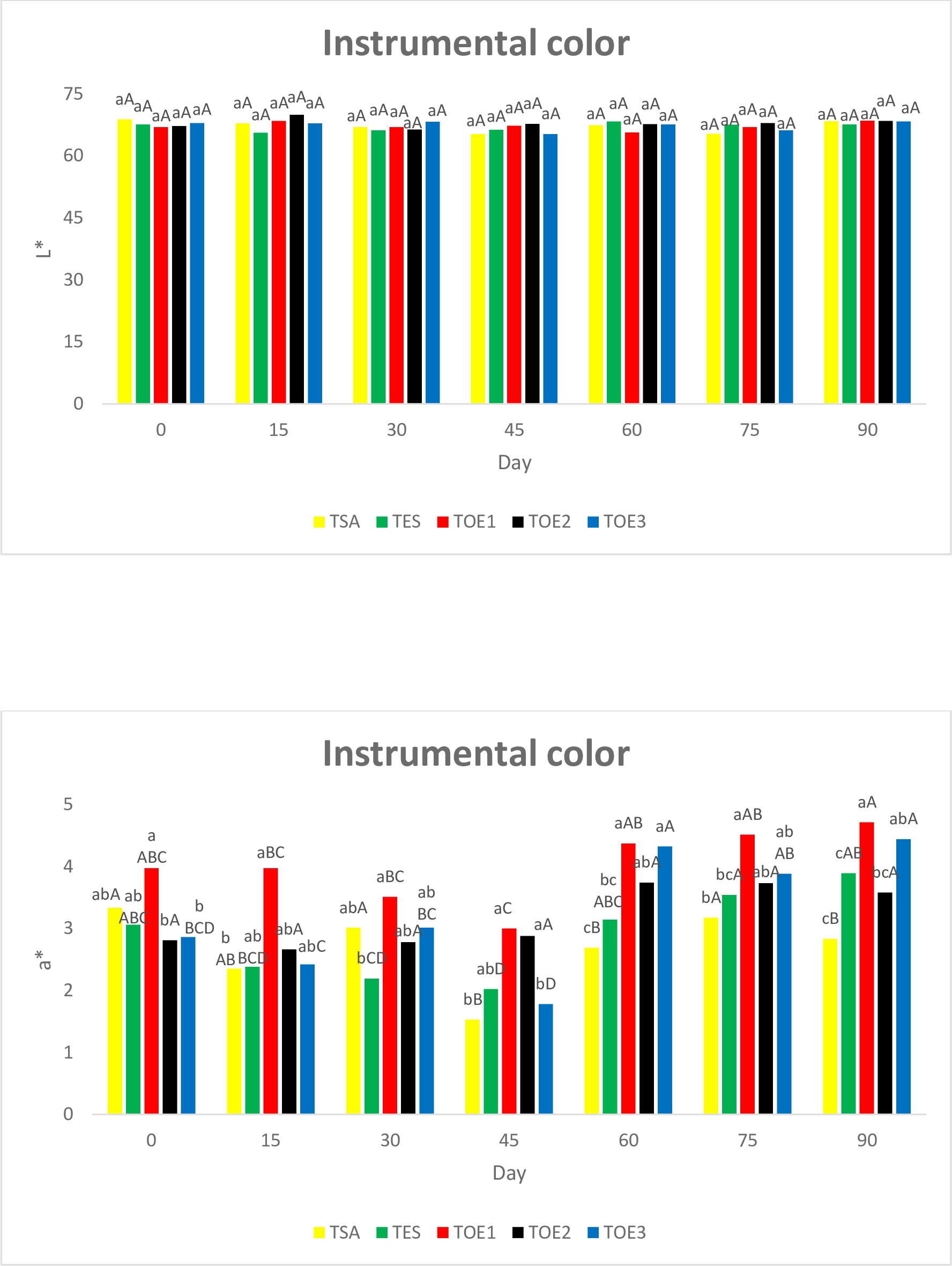

Supplement: S1 Fig — (TIF) [file pone.0296106.s001.tif]

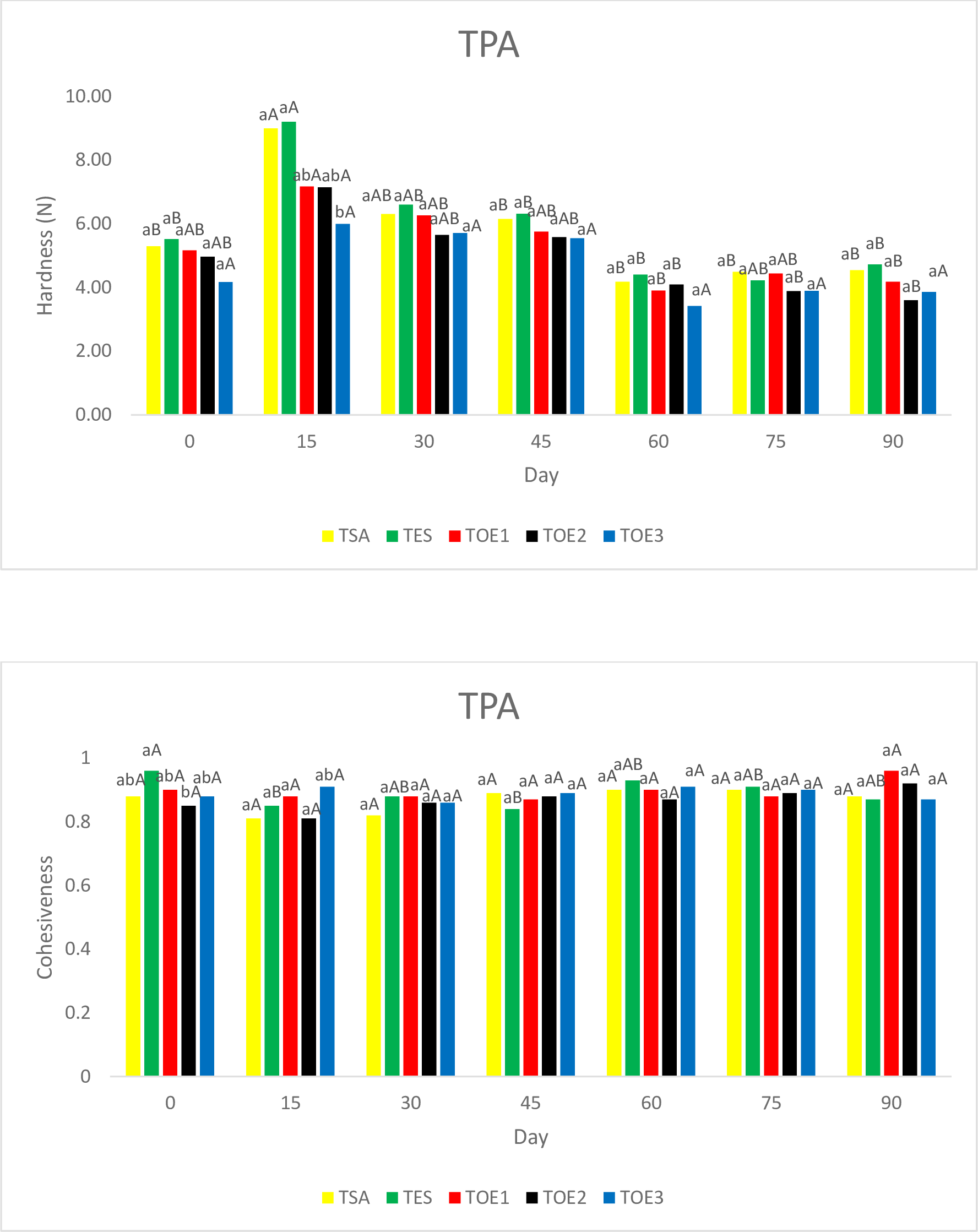

Supplement: S2 Fig — (TIF) [file pone.0296106.s002.tif]
