# Supplementary material for: Use of Nile tilapia (Oreocromis niloticus) processing residues in the production of pâtés with the addition of oregano (Origanum vulgare) essential oil
Source: PLoS One. 2023 Dec 18;18(12):e0296106. doi: 10.1371/journal.pone.0296106 (PMC10727447; doi:10.1371/journal.pone.0296106)
Supplement: S1 Table — (DOCX) [file pone.0296106.s003.docx]

**S1 Table. Microbiological analysis of tilapia pâtés with the addition of oregano essential oil.**

| **Parameters** | **Day** | **TSA** | **TES** | **TOE1** | **TOE2** | **TOE3** |
| --- | --- | --- | --- | --- | --- | --- |
| Coagulase -positive staphylococci (CFU g^-1^) | 0 | <1x10^2^ | <1x10^2^ | <1x10^2^ | <1x10^2^ | <1x10^2^ |
|  | 30 | <1x10^2^ | <1x10^2^ | <1x10^2^ | <1x10^2^ | <1x10^2^ |
|  | 60 | <1x10^2^ | <1x10^2^ | <1x10^2^ | <1x10^2^ | <1x10^2^ |
|  | 90 | <1x10^2^ | <1x10^2^ | <1x10^2^ | <1x10^2^ | <1x10^2^ |
| Psychrotrophic  (CFU g^-1^) | 0 | <1x10^2^ | <1x10^2^ | <1x10^2^ | <1x10^2^ | <1x10^2^ |
|  | 30 | <1x10^2^ | <1x10^2^ | <1x10^2^ | <1x10^2^ | <1x10^2^ |
|  | 60 | <1x10^2^ | <1x10^2^ | <1x10^2^ | <1x10^2^ | <1x10^2^ |
|  | 90 | <1x10^2^ | <1x10^2^ | <1x10^2^ | <1x10^2^ | <1x10^2^ |
| Total coliforms (MPN g^-1^) | 0 | <1x10^2^ | <1x10^2^ | <1x10^2^ | <1x10^2^ | <1x10^2^ |
|  | 30 | <1x10^2^ | <1x10^2^ | <1x10^2^ | <1x10^2^ | <1x10^2^ |
|  | 60 | <1x10^2^ | <1x10^2^ | <1x10^2^ | <1x10^2^ | <1x10^2^ |
|  | 90 | <1x10^2^ | <1x10^2^ | <1x10^2^ | <1x10^2^ | <1x10^2^ |
| *Salmonella* sp. | 0 | absence | absence | absence | absence | absence |
|  | 30 | absence | absence | absence | absence | absence |
|  | 60 | absence | absence | absence | absence | absence |
|  | 90 | absence | absence | absence | absence | absence |

Treatments: TSA - control treatment; TES with the addition of sodium erythorbate; and formulation TOE1 with 600 ppm of oregano essential oil; TOE2 with 1000 ppm of essential oil; and TOE3 with 1400 ppm of essential oil.
